# Immunohistochemical evaluation of human epidermal growth factor receptor 2 and estrogen and progesterone receptors in breast carcinoma in Jordan

**DOI:** 10.1186/bcr1200

**Published:** 2005-05-24

**Authors:** Nidal M Almasri, Mohammad Al Hamad

**Affiliations:** 1 Department of Pathology, Jordan University of Science and Technology, and King Abdullah University Hospital, Irbid, Jordan

## Abstract

**Introduction:**

Although breast carcinoma (BC) is the most common malignancy affecting Jordanian females and the affected population in Jordan is younger than that in the West, no information is available on its biological characteristics. Our aims in this study are to evaluate the expression of estrogen receptor (ER) and progesterone receptor (PR) and Her-2/neu overexpression in BC in Jordan, and to compare the expression of these with other prognostic parameters for BC such as histological type, histological grade, tumor size, patients' age, and number of lymph node metastases.

**Method:**

This is a retrospective study conducted in the Department of Pathology at Jordan University of Science and Technology. A confirmed 91 cases of BC diagnosed in the period 1995 to 1998 were reviewed and graded. We used immunohistochemistry to evaluate the expression of ER, PR, and Her-2. Immunohistochemical findings were correlated with age, tumor size, grade and axillary lymph node status.

**Results:**

Her-2 was overexpressed in 24% of the cases. The mean age of Her-2 positive cases was 42 years as opposed to 53 years among Her-2 negative cases (p = 0.0001). Her-2 expression was inversely related to ER and PR expression. Her-2 positive tumors tended to be larger than Her-2 negative tumors with 35% overexpression among T3 tumors as opposed to 22% among T2 tumors (p = 0.13). Her-2 positive cases tended to have higher rates of axillary metastases, but this did not reach statistical significance. ER and PR positive cases were seen in older patients with smaller tumor sizes.

**Conclusion:**

Her-2 overexpression was seen in 24% of BC affecting Jordanian females. Her-2 overexpression was associated with young age at presentation, larger tumor size, and was inversely related to ER and PR expression. One-fifth of the carcinomas were Her-2 positive and ER negative. This group appears to represent an aggressive form of BC presenting at a young age with large primary tumors and a high rate of four or more axillary lymph node metastases.

## Introduction

According to data from the Jordan National Cancer Registry [1], breast carcinoma is the most common malignant neoplasm affecting Jordanian females. Others as well as our own group have shown that females with breast carcinoma in Jordan are significantly younger than those in the West, with an average age ranging from 44.5 to 47 years [2-5]. Such data may reflect the fact that the population of Jordan is, on average, younger than in the West, but it may also suggest that breast carcinoma in Jordan has some unique biological features that need to be explored. Estrogen receptor (ER), progesterone receptor (PR) and human epidermal growth factor receptor 2 (Her-2) expression are crucially important in the biology of breast carcinoma, and yet the expression of these have not been studied in breast carcinoma in Jordan. It is known that ER and PR expression are the only predictive factors with proven usefulness in selecting patients who are likely to respond to adjuvant endocrine therapy [6,7]. Patients lacking these receptors tend to have shorter disease free survival and earlier recurrences than those expressing these receptors [6]. Her-2, otherwise known as neu or c-erbB-2, is the product of an oncogene amplified and overexpressed in 20% to 30% of breast carcinomas [8-13]. In most studies, overexpression of Her-2 is associated with adverse prognosis independent of other prognostic factors, even when multivariate analysis was used for the outcome analysis [14]. The prognostic effects of Her-2 expression appear to be stronger in node positive carcinomas than in node negative carcinomas [15]. Although Her-2 expression in breast carcinomas is associated with resistance to regimens using cyclophosphamide and methotrexate [16], it is associated with better response to regimens using doxorubicin [17]. Her-2 has gained an even greater deal of attention lately after the introduction of a humanized monoclonal antibody known as trastuzumab that can be effective in the treatment of cases in which this oncogene product is overexpressed [18-23].

Our aims in this study are to evaluate the expression of ER and PR and the overexpression of Her-2/neu in patients with breast cancer in Jordan, and to compare the expression of these with other prognostic parameters for mammary carcinomas, such as histological type, histological grade, tumor size, patients' age and number of lymph node metastases.

## Materials and methods

This is a retrospective study of breast carcinoma cases diagnosed in the period January 1995 to December 1998 at the Department of Pathology, Jordan University of Science and Technology (JUST) in Irbid, Jordan. The Department of Pathology at JUST is the sole provider of histopathology services in the north of Jordan. Only cases among Jordanian females with adequate tissue to perform immunhistochemical examination were included. Of 135 cases of breast carcinomas diagnosed in this period, 91 (67%) fulfilled these criteria. The cases reflect the overall distribution of all breast carcinomas diagnosed in the same period of time.

Sections from the cases were reviewed by one of us (NMA), and the tumors were typed according to the WHO classification system [24]. For invasive ductal carcinomas, the Nottingham combined histologic system was used for grading [25]. Grade 1 carcinoma includes tumors with combined scores of 3, 4 or 5; grade 2 includes scores of 6 and 7; and grade 3 includes tumors with the scores of 8 and 9.

Sections were cut at 4 μm thicknesses, mounted onto silanized slides, and left to dry overnight at 37°C. Sections were then deparaffinized and rehydrated. Antigen retrieval was achieved by heat retrieval using a bench autoclave. Briefly, slides were placed in Coplin jars containing enough 0.01 M sodium citrate solution (pH 6.0) to cover the sections, then autoclaved at 121°C for 10 minutes in the case of Her-2, and 15 minutes for both ER and PR. Slides were incubated with 100–200 μl of primary antibodies for 30 minutes at room temperature in a moisture chamber, then rinsed in PBS. The dilution of the primary antibodies against ER and PR (Biogenex, San Ramon, Ca, USA) was 1:130, and for Her-2/neu (Dako, Carpintera, Ca, USA) 1:50. After washing, binding of antibodies was detected by incubation for 10 minutes with biotinylated goat anti-mouse antibody ready to use (LSAB2) from Dako; the slides were then rinsed with PBS. Sections were then incubated with streptavidin-horse radish peroxidase for 10 minutes. Finally, the sections were washed in 4 times in 4 minute changes of PBS, followed by adding 3,3 diaminobenzidine tetra hydrochloride (Biogenex) as a chromogen to produce the characteristic brown stain.

For each run of staining, a positive and negative control slide were also prepared. The positive control slides were prepared from breast carcinoma known to be positive for the antigen under study. The negative control slides were prepared from the same tissue block, but incubated with PBS instead of the primary antibody.

A semi-quantitative histochemical score was used to record results of ER and PR staining according to the system established by Allred *et al*. [7]. This system considers both the proportion and intensity of stained cells. The intensity score (IS) ranges from 0 to 3, with 0 being no staining, 1 weak staining, 2 intermediate staining, and 3 intense staining. The proportion score (PS) estimates the proportion of positive tumor cells and ranges from 0 to 5, with 0 being non-reacting, 1 for 1% reacting tumor cells, 2 for 10% reacting tumor cells, 3 for one-third reacting tumor cells, 4 for two-thirds reacting tumor cells, and 5 if 100% of tumor cells show reactivity. The PS and IS are added to obtain a total score (TS) that ranges from 0 to 8. Tumor cells with a total score of 3 to 8 were considered positive, whereas those with a TS less than 3 were considered negative cases.

**Figure 1:**
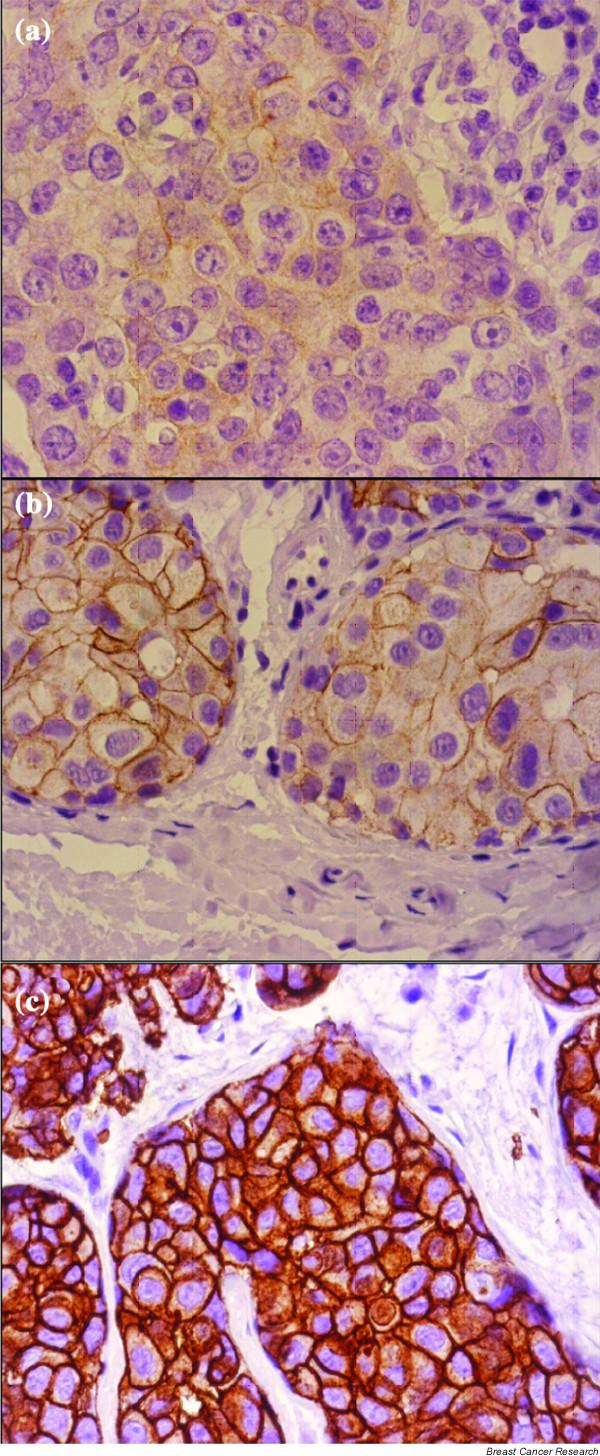
Microscopy pictures illustrating the patterns of Her-2 immunostaining in breast carcinoma. **(a) **Weak positive (1+) pattern exemplified by weak focal membrane staining seen in more than 10% of the tumor cells. **(b) **Intermediate (2+) pattern, showing weak to moderate complete membrane staining in more than 10% of the tumor cells. **(c) **Strongly positive (3+) pattern shows intense membrane staining with weak to moderate cytoplasmic reactivity in more than 10% of the tumor cells. Her-2 overexpressing cases comprised only those having strongly positive (3+) patterns of immunostaining.

Her-2/neu was scored on a 0 to 3 scale according to the criteria set by Dako. The staining was scored as: negative (0) when no membrane staining was observed, or when membranous staining was observed in less than 10% of the tumor cells; weak positive (1+) if weak focal membrane staining was seen in more than 10% of the tumor cells; intermediate (2+) if weak to moderate, complete membrane staining was seen in more than 10% of the tumor cells; and strongly positive (3+) if intense membrane staining with weak to moderate cytoplasmic reactivity was seen in more than 10% of the tumor cells. Figure 1 illustrates scores 1+, 2+, and 3+ as uses in this study. In the final analysis, however, scores 0 and 1 were considered negative; score 2 was considered weakly positive; and score 3 was considered strongly positive. Only score 3 cases were considered as Her-2 overexpressing cases. Fluorescence *in situ *hybridization (FISH) was not performed on the weak positive cases (score 2) in this study.

The Student's t-test was used for comparison of mean tumor size and mean patient age for each category of cases. The chi square test was used to compare the expression of ER, PR and Her-2 among different cases, including: those above or below 50 years of age; those with tumor size up to 2 cm with those between 2 and 5 cm and those larger than 5 cm in size; and those with up to 3 nodal metastases versus those with more than 3 nodal metastases. The results were considered statistically significant if the P value was < 0.05.

## Results

Of the 91 cases included in this study, infiltrating ductal carcinoma (IDC) was the largest group, accounting for 84% (76/91) of all the cases. This group included three mixed ductal and lobular cases and two cases with associated Paget's disease. The second largest group, composed of seven cases, was lobular carcinoma. The remaining cases included five cases of mucinous carcinoma, one case of ductal carcinoma *in situ*, one case of infiltrating ductal carcinoma with medullary features, and one case of metaplastic carcinoma. Among the infiltrating ductal carcinomas, there were only 3 grade 1 cases, 34 grade 2 cases, 37 grade 3 cases, and 2 cases were not graded. The median age of all the cases was 47.5 years ranging from 20 to 75 years. Fifty (57%) of 88 cases with known age were below 50 years of age, whereas 38 cases were 50 years or older.

The mean age of patients with positive Her-2 expression was 42 years (Table 1), which is 10 years younger than those who lack Her-2 expression. This difference is statistically significant (P = 0.0007, Student's t-test). Similarly, Her-2 expression was seen in 34% of patients less than 50 years of age as opposed to 13% in patients 50 years or older (P = 0.003, chi square test).

**Table 1:** Her-2 status and estrogen and progesterone receptor expression in female breast carcinoma patients in Jordan

	Her-2 weak positive	Her-2 strong positive	Her-2 negative	ER positive	ER negative	PR positive	PR negative
Mean age (years)^a^	43.7	42.3	53.2	52.40	43.68	50.74	45.71
No. of patients below 50 years of age	15	17	18	21	29	24	26
No. of patients 50 years of age or older	5	5	28	26	12	22	16
Mean size (cm)^b^	4.9	4.72	4.02	3.57	5.3	4.06	4.79
No. of T1(up to 2 cm) tumors	4	4	7	9	6	8	7
No. of T2 (more than 2 and up to 5 cm) tumors	11	11	29	29	22	29	22
No. of T3 (more than 5 cm) tumors	6	7	7	6	14	7	13
No lymph node metastases^c^	1	3	3	2	5	3	4
1–3 lymph node metastases	3	5	8	7	9	10	6
More than 3 lymph node metastases	9	10	8	13	14	13	14

^a^Age was unknown for three cases; ^b^size was unknown for five cases;

^c^determination of axillary lymph node status was limited to 50 patients who underwent lymph node dissection.

The mean age of patients with positive ER expression was 52.4 years as opposed to 43.7 among patients lacking ER expression (P = 0.001, Student's t-test). For patients less than 50 years of age 42% were ER negative, whereas 68% of patients 50 years or older were ER positive (P = 0.009, chi square test).

The mean age for PR negative cases was 45.7 years and the mean age for PR positive cases was 50.7 years (P = 0.06, Student's t-test). PR positive patients comprised 48% of patients less than 50 years of age compared to 58% of patients 50 years old or older (P = 0.36, chi square test).

Tumors with strong Her-2 expression tended to be larger than those lacking expression (scores 0 and 1), with mean sizes of 4.7 cm and 4 cm, respectively. Among patients with tumor size more than 5 cm (T3), 35% were Her-2 positive (3+) compared to 22% with tumors more than 2 and up to 5 cm in size (T2) (P = 0.13, Chi square test).

The mean size of tumors expressing ER was 3.6 cm versus 5.3 cm for those lacking ER expression (P = 0.009, Student's t-test). ER positive tumors comprised 57% of tumors between 2 and 5 cm but only 30% of those larger than 5 cm (P = 0.04, chi square test).

For PR positive cases, the mean tumor size was 4.1 cm, and the average size for PR negative cases was 4.8 cm. (P = 0.3, Student's t-test). PR positive tumors comprised 57% of tumors between 2 and 5 cm compared to 35% of those larger than 5 cm (P = 0.1, chi square test).

Lymph node status was known in 50 (55%) of the 91 cases included in this study. As shown in Table 1, 56% of the Her-2 positive cases had more than three lymph node metastases, as opposed to 42% among the Her-2 negative cases (P = 0.29, chi square test).

The fraction of ER positive cases among those with up to three lymph node metastases was 39%, slightly lower than the 48% seen among those with more than three lymph node metastases, but this difference was not statistically significant. Similarly, no correlation between lymph node metastases and PR expression was detected.

Grading analysis was limited to 74 of the 76 cases of IDC as 2 cases were not graded. Her-2 overexpression was seen in similar proportions of grade 2 and 3 breast carcinomas, 26.4% and 27% of the cases, respectively. Similarly, no correlation was detected between the grade of the tumors and expression of ER and PR (P values 0.76 and 0.32, respectively).

A negative correlation between Her-2 expression and ER and PR was noted. Of the 22 Her-2 positive cases, 82% were ER negative. On the other hand, 42% of ER negative carcinomas were Her-2 positive. A negative correlation was also seen between Her-2 and PR expression. Of the PR negative carcinomas, 68% were Her-2 positive, and 65% of Her-2 negative cases were PR positive.

On the other hand, a positive correlation between ER and PR was detected. Of the 48 ER positive cases, 36 (75%) were PR positive. Similarly, 32 (74%) of the ER negative were also PR negative.

We noticed that two distinct groups of carcinomas can be distinguished: group 1 includes those with negative Her-2 and positive ER; and group 2 includes those with positive Her-2 and negative ER (Table 2). Group 1 had a relatively much smaller tumor size (3.6 cm) and an older mean age of 55 years at the time of diagnosis. Group 2 had an average tumor size of 4.9 cm and a mean age of 41 at the time of diagnosis. The difference between these groups was statistically highly significant.

**Table 2:** Her-2 negative and ER positive compared to Her-2 positive and ER negative breast carcinoma cases

	Her-2 negative and ER positive	Her-2 positive and ER negative	P value
No. of patients	35	18	-
Mean age (years)	54.88	41.2	0.0003
Mean tumor size (cm)	3.6	4.9	0.088
No. of patients without axillary node dissection	21	8	0.002
No. of patients who underwent axillary nodes dissection	14	23	
No. of patients with up to 3 lymph node metastases	8	11	0.48
No. of patients with more than 3 lymph node metastases	6	12	

## Discussion

Breast carcinoma is a disease with a tremendous heterogeneity in its clinical behavior. Clinical and pathological variables such as tumor size, histologic grade, histologic type, lymph node metastases, vascular space invasion, tumor cell proliferation, tumor necrosis, extent of ductal carcinoma *in situ*, age, and pregnancy may help in predicting prognosis and the need for adjuvant therapy [24]. Newer prognostic factors and predictors of response to therapy are needed, however, to distinguish subgroups with different biological features within carcinomas that otherwise appear homogenous according to classic pathological and clinical criteria. ER, PR and Her-2 represent the most acceptable factors for predicting prognosis, response or resistance to treatment, and the potential use of newer drugs such as trastuzumab in the case of Her-2 overexpression.

In this study, we found that 22 (24%) of 91 cases were Her-2 positive. Although there is a wide variation in Her-2 overexpression and amplification, our figure appears to be within the commonly accepted rate of 20% to 30% [9,11-13,26,27]. It does appear, however, to be lower than those reported in East Asia [28,29] and in neighboring countries such as Lebanon [30] and Egypt [31]. Her-2 was expressed in 28% of the infiltrating ductal carcinoma cases compared to only 14% of our seven lobular carcinoma cases. This pattern of low Her-2 expression in lobular carcinoma is in agreement with data reported in the literature [32-35]. None of the other types of breast carcinoma showed evidence of Her-2 expression.

We found a clear negative correlation between Her-2 overexpression and age in this study. The mean age of Her-2 positive patients was 11 years less than those patients lacking Her-2 expression, a statistically significant difference. Similarly, patients younger than 50 years of age were 2.6 times more likely to overexpress Her-2 than patients 50 years of age or older (34% versus 13%). It should be pointed out that higher rates of Her-2 overexpression in young patients have been documented in previous studies [31,36,37]. Our results show a tendency of Her-2 overexpression to be more associated with larger tumor size. Tumors expressing Her-2 were on the average 0.7 cm larger than those lacking Her-2 expression, although this difference was not statistically significant. Similarly, the fraction of tumors larger than 5 cm tended to have higher rates of Her-2 expression than those 2 to 5 cm in size (35% versus 22%), but this difference was not statistically significant (P = 0.13).

Other groups have shown a direct relationship between lymph node metastases and Her-2 expression [32,38-40]. Our data reveal that 56% of Her-2 overexpressing tumors had more than three lymph node metastases, as opposed to 42% of Her-2 negative cases, although this difference was not statistically significant. We believe that the low number of cases with known nodal status is responsible for the lack of significant correlation in this study; therefore, future studies with larger numbers of patients are needed to confirm the association of Her-2 expression with nodal metastases. Similarly, we were unable to show a significant relationship between Her-2 expression and the histologic grade of breast carcinoma. Other studies concluded that Her-2 expression or amplification is associated with grade [32,36,40,41]. It should be pointed out, however, that the low number of grade 1 carcinomas (three cases) in this study would not allow us to evaluate this variable with any degree of confidence.

ER was expressed in 53% of our cases. This figure is less than the number of ER positive cases reported in the literature (60% to 70%) [7]; however, there is a wide variation in ER expression and our figure would probably fall in the lower reported ranges [42-46]. In our study, there is a strong correlation between patient age and ER expression. The mean age for ER expressing carcinoma patients was nine years older than those lacking ER expression (P = 0.001). Similarly, 68% of patients 50 years or older were ER positive as opposed to 42% of patients less than 50 years old; this difference was statistically significant (P = 0.01). These findings are in agreement with other reports in the literature, which show an association between ER expression in breast carcinoma patients and age at the time of diagnosis [42,47,48]. In our cases, we also found that ER expressing breast carcinomas were, on average, 1.6 cm smaller than carcinomas lacking ER expression (P = 0.009). Similarly, 57% of T2 tumors (2 to 5 cm) were ER positive as opposed to only 30% of T3 tumors (P = 0.04). These figures are also in agreement with data reported in the literature [38,43,49]. Our data also show a strong inverse correlation between Her-2 and ER expression, which is in agreement with data reported by others [10,39,50,51]. Unlike reported data that shows a correlation between ER expression and tumor grade [41,43,47,49], however, we were unable to confirm such a correlation in our cases. The lack of association between ER expression and lymph node status in our study supports the findings of Chariyalertsak *et al*. [50] who found no correlation between ER expression and lymph node status in their breast carcinomas cases.

A strong correlation between ER and PR expression was noted in our series. Unlike ER, however, there was only a low tendency for PR to be associated with smaller carcinomas and with older patients, but this tendency was not statistically significant.

## Conclusion

We have shown for the first time that Her-2 is expressed in approximately one-fourth of breast carcinomas in Jordan. This expression is strongly associated with some known bad pathological and clinical prognostic factors, such as young age, large tumor size and lack of ER and PR expression. In contrast, ER expression was seen in older patients, and was associated with small tumor size and lack of Her-2 expression. One-fifth of the breast carcinoma cases in this study have a profile characterized by overexpression of Her-2 and lack of ER expression. This subgroup of patients appears to represent an aggressive form of breast carcinoma characterized by young age presentation (median 40 years), large tumor size (5.4 cm), and high rates of axillary lymph node metastases (four or more).

## Abbreviations

BC = breast carcinoma; ER = estrogen receptor; Her-2 = human epidermal growth factor receptor 2; IS = intensity score; JUST = Jordan University of Science and Technology; PBS = phosphate-buffered saline; PR = progesterone receptor; PS = proportion score; TS = total score.

## Competing interests

The authors declare that they have no competing interests.

## Authors' contributions

NMA designed the study, reviewed the histopathology of breast carcinomas and graded all the cases histologically, interpreted the results of Her-2, ER and PR expression, and drafted the manuscript. MH prepared the histological slides and carried out the immunoperoxidase stains on the cases.
